# Dihydrotestosterone regulating apolipoprotein M expression mediates via protein kinase C in HepG2 cells

**DOI:** 10.1186/1476-511X-11-168

**Published:** 2012-12-05

**Authors:** Ye Yi-zhou, Cao Bing, Li Ming-qiu, Wang Wei, Wang Ru-xing, Rui Jun, Wei Liu-yan, Jing Zhao-hui, Ji Yong, Jiao Guo qing, Zou Jian

**Affiliations:** 1 Department of Cardiovascular Surgery, Affiliated Shanghai 1st People’s Hospital, Shanghai Jiaotong University, Shanghai, 210008, People's Republic of China; 2 Department of Cardiothoracic Surgery, Affiliated Taixing People’s Hospital, Yangzhou Medical University, Taixing, 225400, People's Republic of China; 3 Department of Cardiovascular Surgery, Affiliated Wuxi People’s Hospital, Nanjing Medical University, Qingyang Road 299, Wuxi City, Jiangsu Province, 214023, China; 4 Department of Cardiovascular Surgery, National Center for Cardiovascular Disease, Beijing, 200000, People's Republic of China

**Keywords:** Androgen, DHT, ApoM, PKC

## Abstract

**Background:**

Administration of androgens decreases plasma concentrations of high-density lipid cholesterol (HDL-C). However, the mechanisms by which androgens mediate lipid metabolism remain unknown. This present study used HepG2 cell cultures and ovariectomized C57BL/6 J mice to determine whether apolipoprotein M (ApoM), a constituent of HDL, was affected by dihydrotestosterone (DHT).

**Methods:**

HepG2 cells were cultured in the presence of either DHT, agonist of protein kinase C (PKC), phorbol-12-myristate-13-acetate (PMA), blocker of androgen receptor flutamide together with different concentrations of DHT, or DHT together with staurosporine at different concentrations for 24 hrs. Ovariectomized C57BL/6 J mice were treated with DHT or vehicle for 7d or 14d and the levels of plasma ApoM and livers *ApoM mRNA* were measured. The mRNA levels of ApoM, ApoAI were determined by real-time RT-PCR. ApoM and ApoAI were determined by western blotting analysis.

**Results:**

Addition of DHT to cell culture medium selectively down-regulated *ApoM mRNA* expression and ApoM secretion in a dose-dependent manner. At 10 nM DHT, the *ApoM mRNA* levels were about 20% lower than in untreated cells and about 40% lower at 1000 nM DHT than in the control cells. The secretion of ApoM into the medium was reduced to a similar extent. The inhibitory effect of DHT on ApoM secretion was not blocked by the classical androgen receptor blocker flutamide but by an antagonist of PKC, Staurosporine. Agonist of PKC, PMA, also reduced ApoM. At 0.5 μM PMA, the *ApoM mRNA* levels and the secretion of ApoM into the medium were about 30% lower than in the control cells. The mRNA expression levels and secretion of another HDL-associated apolipoprotein AI (ApoAI) were not affected by DHT. The levels of plasma ApoM and liver *ApoM mRNA* of DHT-treated C57BL/6 J mice were lower than those of vehicle-treated mice.

**Conclusions:**

DHT directly and selectively down-regulated the level of *ApoM mRNA* and the secretion of ApoM by protein kinase C but independently of the classical androgen receptor.

## Introduction

Men exhibit a higher incidence of cardiovascular diseases than women, and men have lower circulating levels of antiatherogenic high-density lipoprotein cholesterol ((HDL-C). Evidence indicates that the cardiovascular actions of sex steroids are primary factors in mediating this gender-related difference. Because androgen administration lowers HDL-C levels in both genders [1], particularly at supraphysiological plasma concentrations, endogenous androgens, such as testosterone, have been implicated in influencing the lipoprotein profile and risk for cardiovascular diseases (CVD). However, the relationship between androgens and lipid metabolism CVD risk factors is highly complex, and the results of different studies are contradictory [2]. A likely explanation for the complex relationship between androgens and CVD is that androgens affect many risk factors. For example, androgens can increase muscle mass, decrease visceral fat mass in some subjects, improve coronary blood flow, increase mood and motivation (perhaps leading indirectly to health benefits), reduce lipoprotein (a) and leptin, improve insulin sensitivity, and provide other potential benefits, such as anti-inflammatory effects [3]. To understand the role of endogenous and therapeutic androgens in CVD, it will be necessary to identify the mechanisms responsible for the changes in HDL-C levels.

Apolipoprotein M (ApoM) is mainly expressed by hepatocytes and tubular epithelial cells in the kidney and is associated mainly with high-density lipoprotein (HDL) in human plasma [4-7]. Mice deficient in ApoM are impaired in their ability to produce preβ-HDL. Further, overexpression of ApoM in LDL-receptor knockout mice protects against atherosclerosis in mice fed a cholesterol-rich diet. These findings indicate that ApoM is important for preβ-HDL formation and may exert a protective effect on the development and progression of atherosclerosis [8]. Moreover, evidence indicates that ApoM levels are possibly regulated by several cytokines in the in the human HepG2 cell line, which was derived from hepatocellular carcinoma [9-14]. However, the pathophysiological importance of ApoM in humans is still unknown.

The androgen receptor (AR) is expressed in the liver, the primary site of lipoprotein regulation, in which it could conceivably alter the expression of genes controlling HDL metabolism. Apolipoprotein AI (ApoAI) levels are reduced after treatment with androgens, suggesting the decreased synthesis or increased catabolism of this core constituent of HDL particles [15,16]. ApoM, which is one of the main constituents of HDL particles, is involved in HDL metabolism and formation of preβ-HDL. Whether androgens regulate the secretion of apoM and further mediate lipid metabolism remains unknown.

To further understand the possible effect of androgens on ApoM secretion, we investigated the effects of 5-dihydrotestosterone (DHT) on the regulation of ApoM expression by HepG2 cells. DHT is a potent natural androgen that, unlike testosterone, cannot be converted to estradiol by aromatase.

## Results

### Effects of DHT on *ApoM mRNA* levels and secretion of ApoM by HepG2 cells

**Figure 1:**
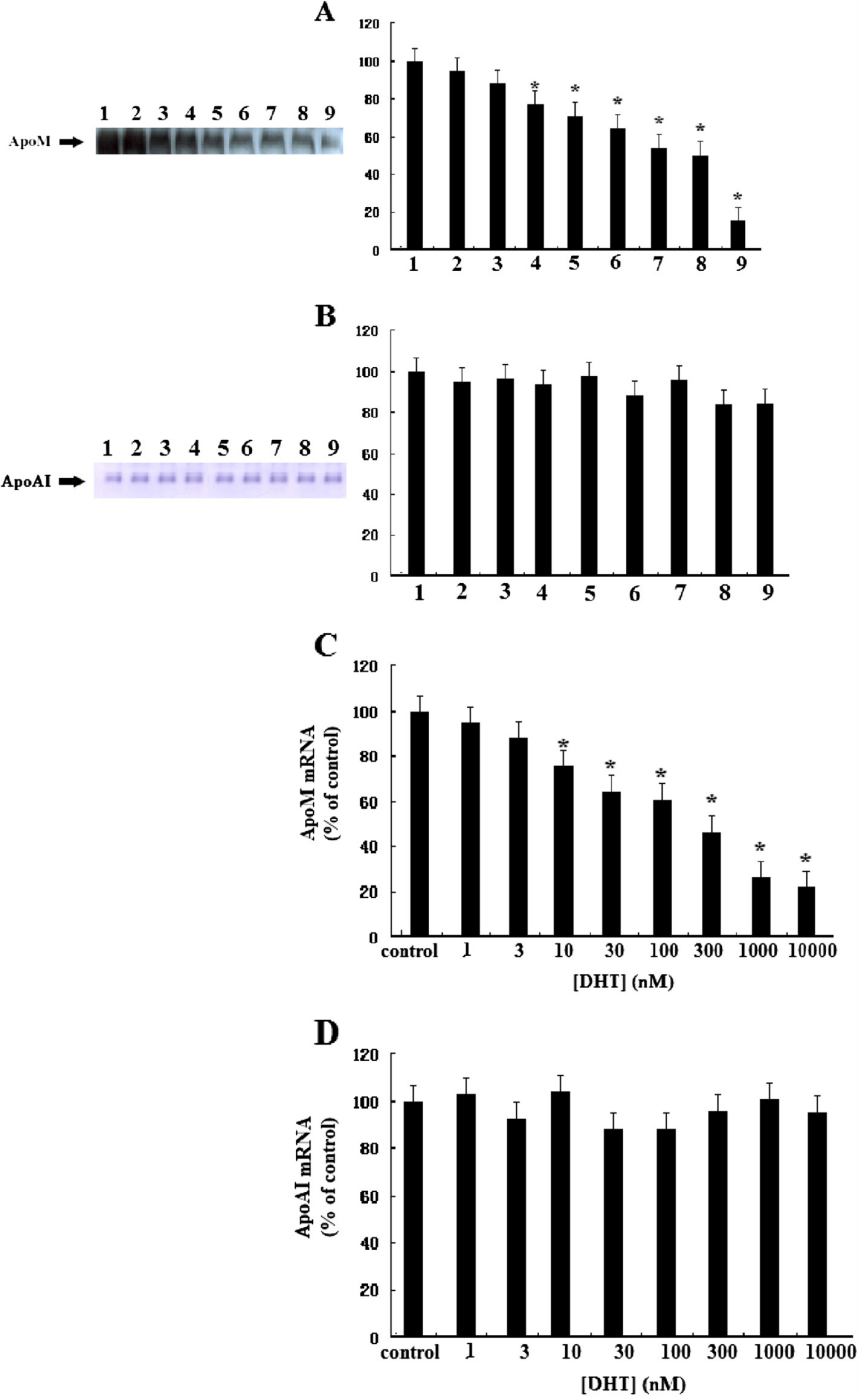
**Effect of DHT on secretion and expression of ApoM and ApoAI.** HepG2 cells were incubated in 2 ml of DMEM containing 10% FBS until subconfluency was achieved. Medium was then changed to 2 ml of medium containing 10% CTFBS and various concentrations of DHT (0, 1, 3, 10, 30, 100, 300, 1000, 10000nM) and incubated for 24 h. ApoM and ApoAI levels were determined by western blotting analysis (**A**, **B**), *ApoM and ApoAI mRNA* levels were determined by RT-PCR (**C**, **D**) as described in “Materials and methods.” Data are expressed relative to the control group (100%). Data are represented as means ± S.D. (n = 6 for each sample group). Lane 1, control group, lanes 2–9, DHT concentrations of 1, 3, 10, 30, 100, 300, 1000 and 10000nM respectively. *P < 0.05 vs. control group.

We first investigated whether DHT could modulate the levels of *ApoM mRNA* and ApoM secretion from HepG2 cells. As shown in Figure 1, DHT significantly inhibited secretion and mRNA levels of ApoM. At 10 nM DHT, ApoM secretion was decreased by 20% (P < 0.05), and at 1000 nM DHT, ApoM secretion was decreased by 60% (P < 0.01) compared with the control media (*A*). To test the specificity of the effect of DHT on ApoM secretion, we determined the effect of DHT on ApoAI secretion in the same cell culture supernatants. DHT did not affect ApoAI secretion at any concentration tested within the levels of detection of the assays (B). DHT also significantly suppressed the levels of *ApoM mRNA* expression in a dose-dependent manner. At 10 nM, the reduction in *ApoM mRNA* was about 20%, and at 1000 nM, it was reduced by more than 70% (P < 0.01) compared with control cells (*C*). However, the levels of *ApoAI mRNA* were not affected by any concentration of DHT (*D*). 

### DHT-suppressed secretion and the mRNA levels of ApoM are not blocked by flutamide

**Figure 2:**
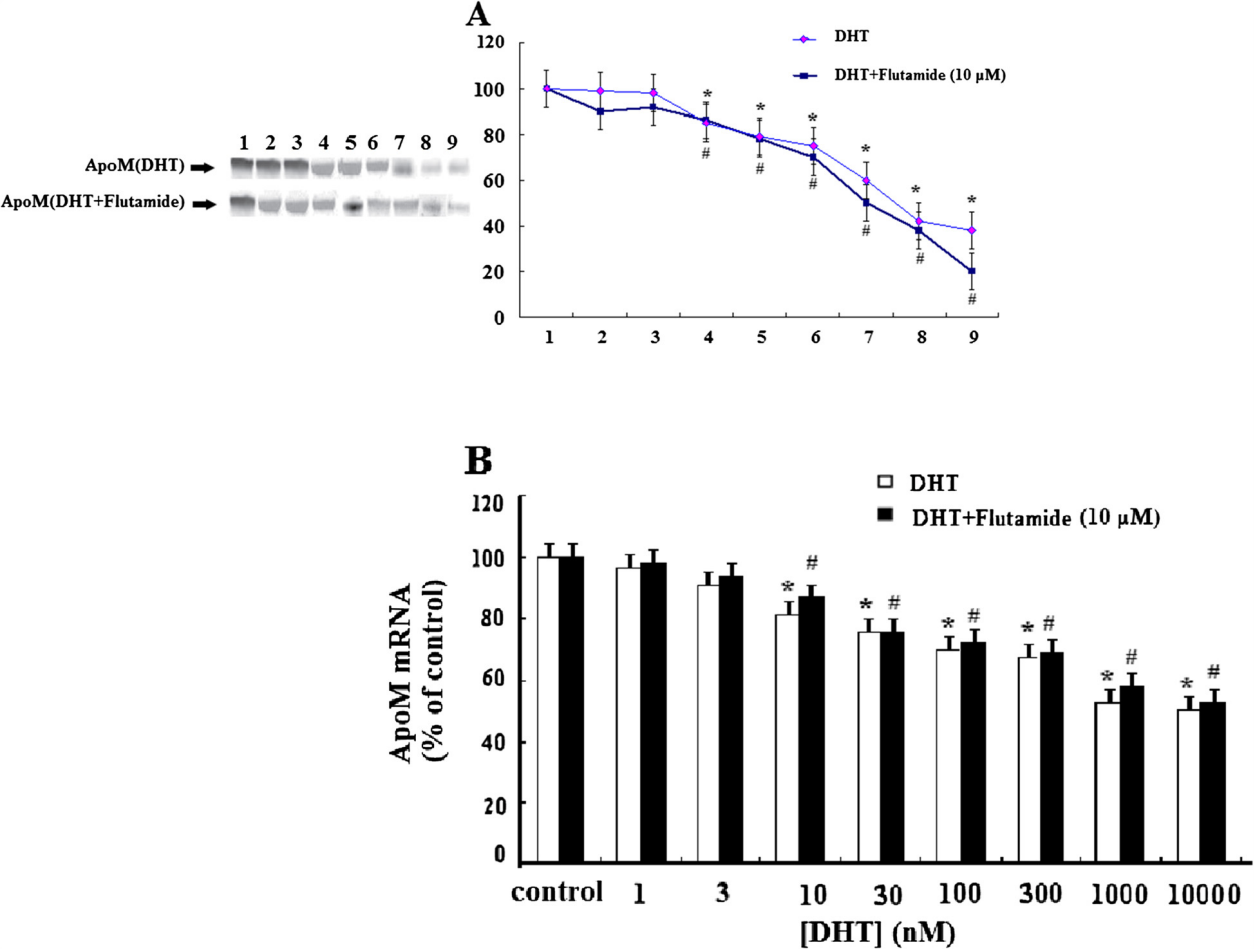
**The effect of DHT on AapoM expression is independent of the classical androgen receptor.** HepG2 cells were treated with 10 μM flutamide or vehicle for 30 min and then incubated in the presence of different concentrations of DHT for 24 h. ApoM concentrations were determined by western blotting analysis (**A**), and *ApoM mRNA* levels were determined by RT-PCR (**B**) as described in “Materials and methods.” Data are expressed relative to the control group (100%). Data are represented as means ± S.D. (n = 6 for each sample group). Lane 1, control group, lanes 2–9, DHT concentrations of 1, 3, 10, 30, 100, 300, 1000 and 10000nM respectively. *,^#^ P < 0.05 versus control group.

To test if the effect of DHT on ApoM secretion and *ApoM mRNA* levels is mediated by the classical androgen receptor, we performed incubations in the presence or absence of the androgen receptor antagonist, flutamide (Figure 2). After 30 min of incubation with flutamide, HepG2 cells were incubated with different concentrations of DHT for 24 h, thereby resulting in the suppression of the secretion of ApoM *(A)* and the levels of *ApoM mRNA (B)* in a dose-dependent manner. This demonstrated that flutamide did not change the effects of DHT on ApoM secretion or *ApoM mRNA* levels, although HepG2 cells express the classical androgen receptor. 

### PKC is involved in DHT-mediated apoM secretion

**Figure 3:**
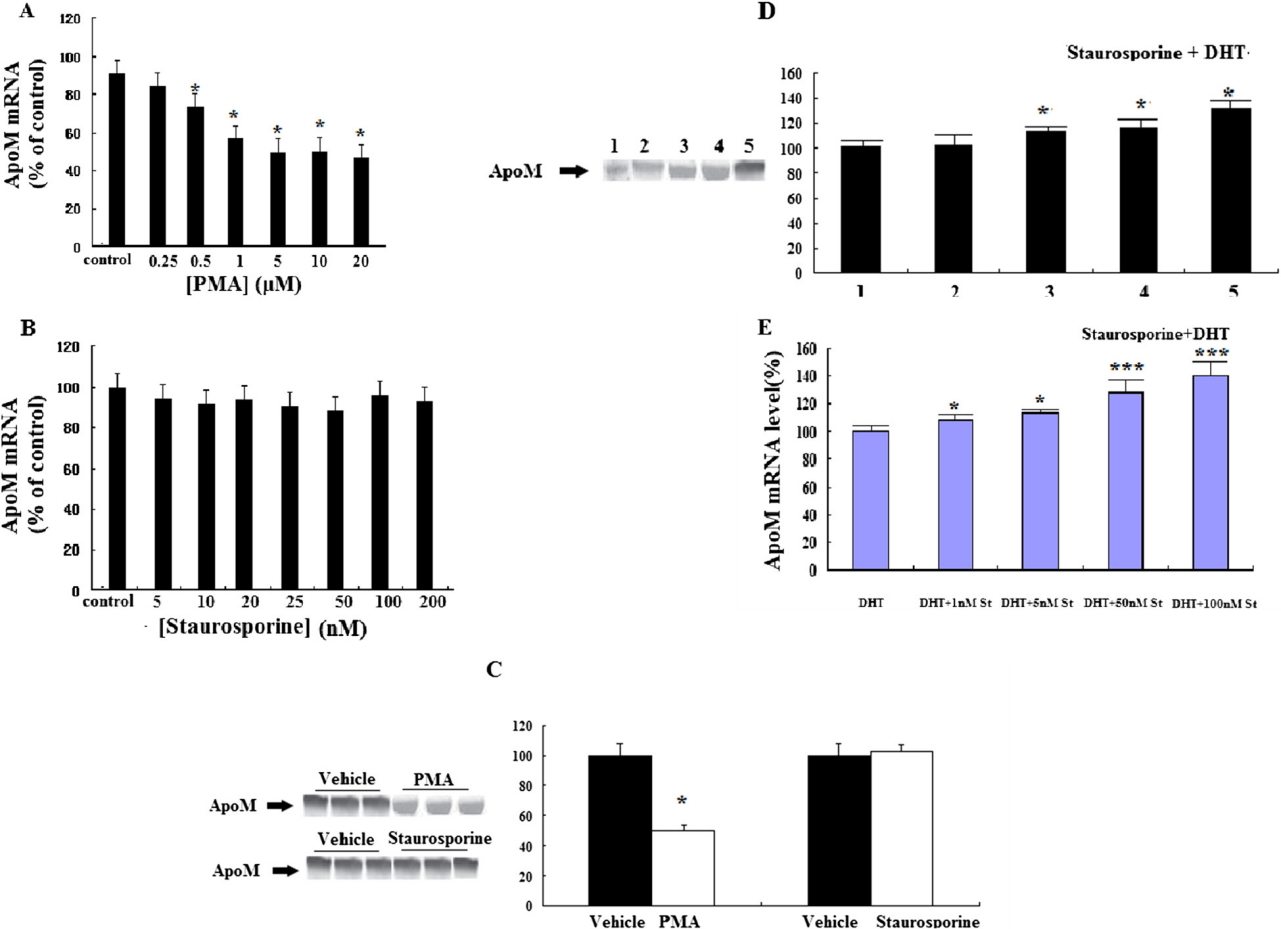
**The effect of DHT on the secretion of ApoM secretion and the levels of *ApoM mRNA *are mediated by PKC pathways.** Cells were treated with different concentrations of PMA or Staurosporine or vehicle (control) for 24 h, the levels of *ApoM mRNA* were determined by RT-PCR (**A**,**B**). Cells were treated with 5 μM PMA or 50nM Staurosporine or vehicle for 24 h, ApoM concentrations were determined by western blotting analysis (**C**). Cells were incubated in 1 μM DHT for 24 h in present different concentrations of Staurosporine or absent Staurosporine (control). ApoM concentrations were determined by western blotting analysis (**D**), Lane 1, control group(1 μM DHT without Staurosporine), lanes 2–5, 1 μM DHT with Staurosporine concentrations of 1,5, 50, and 100 nM respectively. The levels of *ApoM mRNA* were determined by RT-PCR as described in “Materials and methods.” Data are expressed relative to the control group (100%). Data are represented as means (SD) (n = 6 for each sample group). *, P < 0.05 vs. control group. ***, P < 0.01 vs. control group.

The PKC superfamily comprises 9 protein kinases. To determine whether PKC is involved in DHT-mediated ApoM secretion, HepG2 cells were incubated with PMA or Staurosporine in the presence or absence of DHT (Figure 3). PMA decreased the expression and secretion of ApoM (Figure 3A, C). Staurosporine alone had no effect on the levels of ApoM and *ApoM mRNA* (Figure 3B, C). Staurosporine abolished the DHT-mediated decrease in ApoM secretion and expression (Figure 3D, E). These results indicate that PKC affects the DHT-mediated decrease in ApoM secretion and *ApoM mRNA* expression. To determine whether PI3-K is involved in the DHT-mediated reduction of ApoM secretion and the decrease in the levels of its mRNA, HepG2 cells were also incubated with the wortmannin, an inhibitor of PI3-K. The PI3-K inhibitor wortmannin did not detectably alter the effects of DHT on *ApoM mRNA* levels or its secretion (data not shown). 

### Plasma ApoM and hepatic ApoM mRNA levels from mice

**Figure 4:**
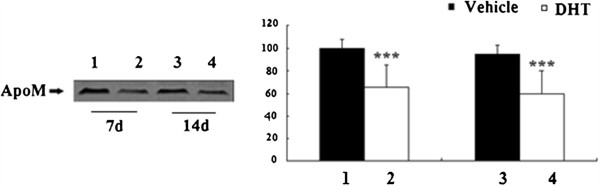
**Effect of DHT on plasma ApoM of DHT-treated C57BL/6 J mice.** C57BL/6 J female mice were ovariectomized at the age of 3 months and treated at the age of 7 months. Animals were randomized into four groups (n = 6), with two groups receiving vehicle (propylene glycol) alone, and two groups receiving 3 mg/kg DHT. All animals were treated daily by sc injections for 7d or 14d, fasted overnight, and killed using CO2. Levels of plasma ApoM were determined by Western blotting as described in “Materials and methods.” Data are expressed relative to the vehicle-treated group (100%). Data are represented as means ± S.D. (n = 6 for each treated group). Lane 1, vehicle-treated mice for 7d, Lane 2, DHT-treated mice for 7d, Lane 3, vehicle-treated mice for 14d and Lane 4, DHT-treated mice for 14d. Significant differences were determined using ANOVA. *P < 0.05 vs. vehicle-treated mice, ***, P < 0.01 vs. vehicle-treated mice.

**Figure 5:**
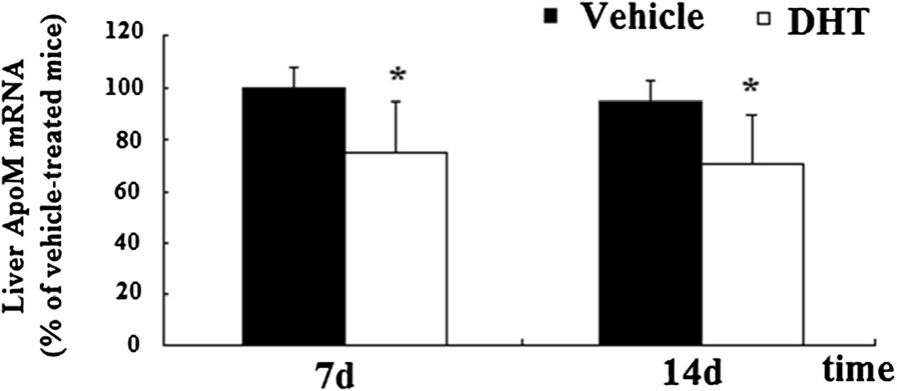
**Effect of DHT on liver ApoM mNRA of DHT-treated C57BL/6 J mice.** C57BL/6 J female mice were ovariectomized at the age of 3 months and treated at the age of 7 months. Animals were randomized into four groups (n = 6), with two groups receiving vehicle (propylene glycol) alone, and two groups receiving 3 mg/kg DHT. All animals were treated daily by sc injections for 7 or 14 d, fasted overnight, and killed using CO2. Livers were frozen in liquid nitrogen for *ApoM RNA* analysis. The levels of *ApoM mRNA* were determined by RT-PCR as described in “Materials and methods.” Data are expressed relative to the vehicle-treated group (100%). Data are represented as means ± S.D. (n = 6 for each treated group). Significant differences were determined using ANOVA. *P < 0.05 vs. vehicle-treated mice, ***, P < 0.01 vs. vehicle-treated mice.

To analyze the effect of androgens on ApoM expression and secretion, levels of plasma ApoM and liver *ApoM mRNA* of DHT-treated mice were measured and compared with those of vehicle-treated mice. Levels of plasma ApoM was analyzed by Western Blot. Levels of plasma ApoM were reduced in DHT-treated mice significantly (Figure 4). Livers were frozen in liquid nitrogen for *ApoM RNA* analysis after mice were killed using CO2. Levels *ApoM mRNA* were measured with RT-PCR. Levels of liver *ApoM mRNA* were reduced in DHT-treated mice significantly (Figure 5). 

## Discussion

In the present study, we demonstrate that ApoM expression is regulated by DHT in HepG2 cells in a dose-dependent manner and that inhibition of ApoM expression by DHT is mediated by PKC. Moreover, DHT did not affect ApoAI expression and we demonstrated further that inhibition of PI3K did not influence DHT-mediated apoM expression.

Androgens administration lowers HDL-C levels in both genders [1], but androgens modulate cholesterol metabolism in poorly understood ways. Most studies indicate that administration of natural or synthetic androgens produces a plethora of effects, some of which appear negative, such as reduction of HDL-C levels, and others positive, such as increased lean mass and reduced visceral fat, lower total cholesterol, and improved sensitivity to insulin [17]. To understand the role of endogenous and therapeutic androgens in CVD, it will be necessary to identify the mechanisms responsible for the reduction in HDL-C. One hypothesis considers reduced synthesis of ApoAI, ApoM, or HDL. The second hypothesis considers that there is decreased donation of cholesterol from peripheral tissues to HDL particles, and the third proposes that there is increased clearance of HDL-C. The fourth, a more complex hypothesis, is that androgens lead to HDL remodeling [15,18], cholesterol redistribution [19], or changes in lipoprotein catabolism.

ApoAI and apoM are constituents of HDL. In some studies, ApoAI levels are reduced after androgen treatment, suggesting decreased synthesis or increased catabolism of HDL [15,16]. ApoM was first identified as a component of human postprandial lipoproteins in 1999 [4]. It was estimated that ~5% of HDL particles contain ApoM [20]. ApoM content in the healthy human plasma pool was 0.94 mM. This roughly corresponds to 1/50^th^ of the mean molar concentration of apoA-I in plasma [21]. However, the physiopathological functions of ApoM are not fully elucidated. Studies of the regulation of ApoM expression may reveal the clinical importance of ApoM.

The AR is highly expressed in adipocytes and regulates their function by a variety of mechanisms, including local transcriptional regulation of lipases and increased levels of adrenergic receptors as well as inhibition of adipogenesis [19]. The AR is also expressed in liver, a primary site of lipoprotein regulation, in which it could conceivably alter the expression of genes controlling HDL metabolism [22]. ApoM is mainly expressed in the hepatocytes. Here, we administered DHT to examine its effect on ApoM secretion and *ApoM mRNA* levels in HepG2 cells. The present study supports the view that DHT affects lipoprotein production by demonstrating that DHT significantly decreased *ApoM mRNA* levels in hepG2 cells and the secretion of ApoM secretion into the medium, whereas there was no effect on the *ApoAI mRNA* levels. These findings suggest that there are different mechanisms for regulating ApoAI and ApoM expression in this particular cell line. To analyze the effect of androgens on ApoM expression and secretion in vivo, we administrated DHT to the ovariectomized C57BL/6 J mice. Plasma levels of ApoM and liver *ApoM mRNA* of DHT-treated mice were measured and compared with those of vehicle-treated mice. It demonstrated that DHT reduced the levels of plasma ApoM and liver *ApoM mRNA* in DHT-treated mice. The present findings, therefore, might partially indicate a mechanism underlying the reduction of plasma HDL cholesterol during administration of DHT in vivo.

Sphingolipids are a large family of glycolipids and phospholipids that share a common sphingoid base backbone. These once called ‘structural’ lipids are now well-established signaling molecules that play multiple roles in a vast number of cellular processes. A growing body of literature has demonstrated the reciprocal interaction between bioactive sphingolipids and steroid hormones. Sphingolipids serve as second messengers in steroidogenic regulatory pathways [23,24], and meanwhile steroid hormones regulates the metabolism of sphingolipids [25-27]. Plasma sphingosine-1-phosphate (S1P), which maintains vascular integrity, is associated with HDL (∼65%) and albumin (∼35%) [28,29]. HDL induced vasorelaxation as well as barrier-promoting and prosurvival actions on the endothelium have been attributed to S1P signaling [30-32]. ApoM is a lipocalin that resides mainly in the plasma HDL fraction [4]. The retained hydrophobic NH2-terminal signal peptide anchors ApoM in the phospholipid layer of the lipoprotein and prevents filtration of the ∼ 22-kDa protein in the kidney [33]. Studies in ApoM gene-modified mice suggest that apoM has antiatherogenic effects, possibly related in part to ApoM’s ability to increase cholesterol efflux from macrophage foam cells, to increased preβ-HDL formation, and to antioxidative effects [8,20,34]. ApoM is a carrier of S1P in HDL and the HDL-associated ApoM–S1P complex mediates vasoprotective actions on the endothelium. This signaling axis may be critical in normal vascular homeostasis and perturbed in vascular diseases [35]. Whether DHT affected HDL-associated function via regulation of ApoM and ApoM–S1P signaling axis is still to be elucidated.

It is well known that androgens exert both transcriptional and non-transcriptional actions [36-38]. The transcriptional actions of androgens are mediated through the classic androgen receptor. The ligand-bound classic androgen receptor mainly functions as a transcription factor modulating the expression of androgen-receptor target genes. In contrast, non-transcriptional actions of androgens include increasing the concentration of intracellular calcium, and activation of protein tyrosine kinase, such as Src(c-Src), extracellular signal-regulated kinase-1/2 (ERK-1/2), and phosphatidylinositol 3-kinase (PI3K) [39-44]. In our present study, we found that flutamide, a classical androgen receptor blocker, did not modify DHT-mediated apoM secretion. Although these data may suggest that the action of DHT on ApoM secretion is non-transcriptional, the differentiation between non-transcriptional vs. transcriptional effects is much more complex and cannot been firmly concluded from the present study.

We also investigated the intracellular signaling mechanisms by which DHT mediates ApoM secretion by hepG2 cells. Our present study shows that PMA, a PKC agonist, increased ApoM secretion. Staurosporin, a PKC superfamily inhibitor, abolished the DHT-mediated decrease in ApoM secretion. The intracellular signaling mechanisms by which DHT act through PKC to affect apoM secretion remains unknown. It is reported that *ApoM gene* expression is affected by nuclear receptors such as hepatocyte nuclear factor-1a (HNF-1a) [45], hepatocyte nuclear factor-4a (HNF-4a), liver receptor homolog-1 (LRH-1) [46], and liver X receptor (LXR) [47].

Leptin is the first identified endocrine product of adipose tissue and was found to regulate vascular function through local and central mechanisms [48]. There is some evidence supporting the effects of leptin on the cardiovascular system and Type 2 diabetes mellitus (T2DM). It was shown that a high leptin level predicts subsequent development of T2DM [49]. Plasma leptin levels positively correlated with TG, Lp (a), Apo-A1, glucose, BMI, insulin resistance (HOMA-IR), SBP and DBP levels and negatively with HDL-C levels in T2DM patients [50,51]. Studies suggest that both leptin and leptin-receptor are essential for ApoM expression in vitro and vivo [9,52]. In the present study we demonstrated that DHT down-regulated the expression and the secretion of ApoM. Whether DHT-affected ApoM expression is mediated by specific nuclear receptors or leptin remains to be investigated.

It has been previously reported that ApoM expression is regulated by PI3-kinase in HepG2-cells [53]. In the present study, we used the PI3-K antagonist (wortmannin) to study DHT-treated HepG2 cells. We found that wortmannin could not abolish DHT-mediated inhibition of ApoM expression, which indicates that PI3-K might not be involved in the DHT-induced inhibition of ApoM expression. Our present results indicate that PKC is involved in DHT-mediated ApoM secretion. However, the participation of PKC family members whose identities remain to be determined.

## Conclusions

DHT directly and selectively down-regulated the level of *ApoM mRNA* and the secretion of ApoM by protein kinase C but independently of the classical androgen receptor.

## Materials and methods

### Materials

The human cell line HepG2, which was derived from hepatocellular carcinoma, was obtained from the American Type Culture Collection (ATCC). Dulbecco's modified Eagle's medium (DMEM) and benzylpenicillin and streptomycin from Gibco (Shanghai, China). Dihydrotestosterone (DHT) and flutamide were purchased from Sigma Chemical Co. Ltd. (Shanghai, China). Staurosporine, PMA and wortmannin were purchased from ENZO (Shanghai, China). Six-well cell culture clusters and 25-cm^2^ vented cell culture flasks were purchased from Costar (Shanghai, China). Fetal bovine serum (FBS) and charcoal-treated fetal bovine serum (CTFBS) were obtained from Invitrogen (Shanghai, China). E.Z.N.A. Total RNA Kit II for total RNA purification was from Omega (Shanghai, China). First strand cDNA synthesis kits were obtained from Invitrogen (Shanghai, China). Taqman Universal PCR Master Mix was purchased from TAKARA BioScience and Technology Company (Dalian, China). The LightCycler real-time RT-PCR System was purchased from Roche Applied Science (Shanghai, China). Rabbit monoclonal antibodies against human ApoM, ApoAI, β-actin, and horseradish peroxidase-conjugated goat polyclonal secondary antibody to rabbit IgG (ab6721) were obtained from Abcam.

### Cell cultures

HepG2 cells were maintained in DMEM with 10% FBS(w/v) in the presence of benzylpenicillin (0.1 iu l^-1^) and streptomycin (0.1 g l^-1^) under standard culture conditions (5% CO_2_, 37°C). Cells were seeded in 25-cm^2^ cell culture flasks or in 6-well cell-culture clusters and allowed to grow to 50–70% confluence. Before the experiment, cells were washed twice with phosphate buffered saline (PBS) and once with DMEM with 10% CTFBS(w/v). When inhibitors were used, they were added in fresh media 30 min prior to adding the other reagents. At the end of the incubation period, media were removed and saved for ApoM and ApoAI assays and the cells for determining *ApoM* and *ApoAI mRNA* levels.

### Effect of the androgen receptor antagonist flutamide on DHT-mediated ApoM secretion and *ApoM mRNA* levels

To evaluate whether the effect of DHT on *ApoM mRNA* levels and the secretion of ApoM from HepG2 cells was mediated via the androgen receptor, cells were incubated in the presence or absence of flutamide. The medium was changed when the cells grew to subconfluence, and flutamide (10 μM) was then added to the media. After 30 min of incubation with flutamide, different concentrations of DHT were added, and the media and cells were harvested 24 h later for determining ApoM or ApoAI levels.

### Effect of protein kinase C or phosphatidylinositol 3-kinase on DHT-mediated ApoM secretion and *ApoM mRNA* levels

To evaluate whether the effect of DHT on ApoM secretion from human HepG2 cells was mediated via protein kinase C (PKC), cells were incubated with agonist or antagonist of PKC in the presence or absence of DHT. The medium was changed at subconfluence, after 30 min of incubation with an antagonist of the PKC superfamily (staurosporine, 50 nM) or agonist of PKC (PMA), varying concentrations of DHT were added, and media and cells were harvested 24 h later for the determination of ApoM or ApoAI levels.

To evaluate whether the effect of DHT on ApoM secreted by HepG2 cells was mediated via phosphatidylinositol 3-kinase (PI3-K), cells were incubated in the presence or absence of an inhibitor of PI3-K (wortmannin). After 30 min of incubation with wortmannin (50 nM), different concentrations of DHT were added, and the media and cells were harvested 24 h later for the determination of ApoM.

### Mice

C57BL/6 J female mice were obtained from the Experimental Animal Center of the Chinese Academy of Sciences (Shanghai, China) and maintained in a 12-h/12-h light/dark cycle with unlimited access to chow and water. Mice were ovariectomized at the age of 3 months and treated at the age of 7 months. Animals were randomized into four groups (n = 6), with two groups receiving vehicle (propylene glycol) alone, and two groups receiving 3 mg/kg DHT. All animals were treated daily by sc injections for 7 d or 14 d, fasted overnight, and killed using CO2. Plasmas were collected for ApoM analysis, and livers were frozen in liquid nitrogen for *ApoM RNA* analysis.

### Extraction of total RNA and real time RT-PCR assays

Total HepG2 RNA of was extracted using the E.Z.N.A. Total RNA Kit II according to the manufacturer’s instructions. For reverse transcription 5 μg total RNA was incubated with 0.5 μg T12VN and Superscript III following the manufacture’s suggested protocol. Human *ApoM* primers (forward: 5^′^-TACCAGCCCTTCTGCACTG-3^′^, reverse: 5^′^-ATCGAGGGAAGAGTGGGG-3^′^) and human *ApoAI* primers (forward: 5^′^-GGCTGTCATCTCTCAGGGAGTTAG-3^′^, reverse: 5^′^-ATTTGAACCTGCCTGACCCTTAG-3^′^) and *β-actin* primers (forward: 5^′^- ACTTACGGTAAATGGCCCG −3^′^, reverse: 5^′^- TAGGGGGCGTACTTGGCATA −3^′^) and mouse *ApoM* primers (forward: 5^′^-CCAAATAGGCTGTCCCAGAA-3^′^, reverse: 5^′^-CGAGTCACTTTCCTGGCTTC-3^′^) were designed with Primer Express software (Applied Biosystems). Quantification of *ApoM mRNA* levels or *ApoAI mRNA* levels is relative to *β-actin mRNA* levels and was performed on a LightCycler in a final volume of 20 μl. Optimal conditions were obtained with 2.0 μl of Taqman Universal PCR Master Mix, 22.5 pmol of both forward and reverse primers and 1 μl of RT product. The thermal cycling conditions for human or mouse *ApoM*, *ApoAI*, and *β-actin* included the following steps: 2 min at 50°C and 1 min 95°C to activate Taq polymerase, 40 cycles of 15 sec at 95°C and 1 min at 60°C. Samples were amplified simultaneously in triplicates in one-assay run. The threshold cycle (CT) is defined as the fractional cycle number at which the reporter fluorescence reaches a certain level. The ratio expression of each gene in experimental vs. control samples was calculated as 2^-(meanΔΔCt)^. Significant differences were determined using ANOVA.

### Apolipoproteins M and AI protein mass determinations

The relative molecular masses of ApoM and ApoAI were determined by western blotting analysis. Cell culture medium containing CTFBS or plasma from mice was fractionated by SDS-polyacrylamide gel electrophoresis, and the proteins were transferred to a nitrocellulose membrane, which was incubated with rabbit monoclonal antibodies and goat polyclonal secondary antibody. Bands corresponding to the different apolipoproteins were visualized using an ECL Plus Western blotting detection system (GE Healthcare Life Science) or using the peroxidase staining method and quantified using Quantity One software.

### Statistical analysis

Results are expressed as means ± S.D. Two groups were compared using Student’s *t*-test, and multiple groups were analyzed by factorial ANOVA followed by Newman-Keuls’ post hoc comparisons. Statistical calculations were performed with Statistical software package version 7.1. Differences were considered significant at P < 0.05.

## Competing interests

The authors declare that they have no competing interests.

## Authors’ contributions

RJ and JY participated in the assay of RT-PCR. JZH and WLY participated in the assay of Western blotting. WRX performed the statistical analysis. JZH and LMQ participated in cell culture. YYZ, CB, ZJ, WW and JGQ participated in the project design. All authors read and approved the final manuscript.

## Author’s information

Cao Bing is a co-first author.

## References

[B1] Somboonporn W Testosterone therapy for postmenopausal women: efficacy and safety Semin Reprod Med 2006 24 2 115 124 10.1055/s-2006-939570 16633985

[B2] Shabsigh R Katz M Yan G Makhsida N Cardiovascular issues in hypogonadism and testosterone therapy Am J Cardiol 2005 96 12B 67M 72M 16387571 10.1016/j.amjcard.2005.10.009

[B3] Malkin CJ Pugh PJ Jones RD Jones TH Channer KS Testosterone as a protective factor against atherosclerosis—immunomodulation and influence upon plaque development and stability J Endocrinol 2003 178 3 373 380 10.1677/joe.0.1780373 12967330

[B4] Xu N Dahlbäck B A novel human apolipoprotein (apoM) J Biol Chem 1999 274 44 31286 31290 10.1074/jbc.274.44.31286 10531326

[B5] Luo G Zhang X Nilsson-Ehle P Xu N Apolipoprotein M Lipids Health Dis 2004 3 21 10.1186/1476-511X-3-21 15461812 PMC523857

[B6] Zhang XY Dong X Zheng L Luo GH Liu YH Ekstrom U Nilsson-Ehle P Ye Q Xu N Specific tissue expression and cellular localization of human apolipoprotein M as determined by in situ hybridization Acta Histochem 2003 105 1 67 72 10.1078/0065-1281-00687 12666989

[B7] Zhang XY Jiao GQ Hurtig M Dong X Zheng L Luo GH Nilsson-Ehle P Ye Q Xu N Expression pattern of apolipoprotein M during mouse and human embryogenesis Acta Histochem 2004 106 2 123 128 10.1016/j.acthis.2003.11.004 15147633

[B8] Wolfrum C Poy MN Stoffel M Apolipoprotein M is required for prebeta-HDL formation and cholesterol efflux to HDL and protects against atherosclerosis Nat Med 2005 11 4 418 422 10.1038/nm1211 15793583

[B9] Xu N Nilsson-Ehle P Hurtig M Ahrén B Both leptin and leptin receptor are essential for apolipoprotein M expression in vivo Biochem Biophys Res Commun 2004 321 4 916 921 10.1016/j.bbrc.2004.06.180 15358114

[B10] Xu N Nilsson-Ehle P Ahrén B Correlation of apolipoprotein M with leptin and cholesterol in normal and obese subjects J Nutr Biochem 2004 15 10 579 582 10.1016/j.jnutbio.2004.03.001 15542348

[B11] Xu N Ekstrom U Nilsson-Ehle P Acth decreases the expression and secretion of apolipoprotein b in hepg2 cell cultures J Biol Chem 2001 276 42 38680 38684 10.1074/jbc.M104659200 11514556

[B12] Xu N Zhang XY Dong X Ekström U Ye Q Nilsson-Ehle P Effects of platelet-activating factor, tumor necrosis factor, and interleukin-1alpha on the expression of apolipoprotein M in HepG2 cells Biochem Biophys Res Commun 2002 292 4 944 950 10.1006/bbrc.2002.6755 11944906

[B13] Xu N Hurtig M Zhang XY Ye Q Nilsson-Ehle P Transforming growth factor-beta down-regulates apolipoprotein M in HepG2 cells Biochim Biophys Acta 2004 1683 1 33 37 10.1016/j.bbalip.2004.04.001 15238217

[B14] Xu N Hurtig M Ekström U Nilsson-Ehle P Adrenocorticotrophic hormone retarded metabolism of low-density lipoprotein in rats, Scand J Clin Lab Invest 2004 64 3 217 222 10.1080/00365510410005730 15222631

[B15] Berg G Schreier L Geloso G Otero P Nagelberg A Levalle O Impact on lipoprotein profile after long-term testosterone replacement in hypogonadal men Horm Metab Res 2002 34 2 87 92 10.1055/s-2002-20521 11972293

[B16] Dickerman RD McConathy WJ Zachariah NY Testosterone, sex hormone-binding globulin, lipoproteins, and vascular disease risk J Cardiovasc Risk 1997 4 5–6 363 366 9865668

[B17] Eckardstein A Wu FC Testosterone and atherosclerosis Growth Horm IGF Res 2003 13 Suppl A S72 S84 12914731 10.1016/s1096-6374(03)00059-5

[B18] Grundy SM Vega GL Otvos JD Rainwater DL Cohen JC Hepatic lipase activity influences high density lipoprotein subclass distribution in normotriglyceridemic men. Genetic and pharmacological evidence J Lipid Res 1999 40 2 229 234 9925651

[B19] De Pergola G The adipose tissue metabolism: role of testosterone and dehydroepiandrosterone Int J Obes Relat Metab Disord 2000 24 Suppl 2 S59 S63 10997611 10.1038/sj.ijo.0801280

[B20] Christoffersen C Nielsen LB Axler O Andersson A Johnsen AH Dahlbäck B Isolation and characterization of human apolipoprotein M-containing lipoproteins J Lipid Res 2006 47 8 1833 1843 10.1194/jlr.M600055-JLR200 16682745

[B21] Jungner I Marcovina SM Walldius G Holme I Kolar W Steiner E Apolipoprotein B and A-I values in 147576 Swedish males and females, standardized according to the World Health Organization-International Federation of Clinical Chemistry First International Reference Materials Clin Chem 1998 44 8 Pt 1 1641 1649 9702950

[B22] Nantermet P Harada S Liu Y Cheng S Johnson C Yu Y Kimme D Holder D Hodor P Phillips R Ray WJ Gene Expression Analyses in Cynomolgus Monkeys Provides Mechanistic Insight into High-Density Lipoprotein-Cholesterol Reduction by Androgens in Primates Endocrinology 2008 149 4 1551 1561 10.1210/en.2007-1151 18187556

[B23] Meroni SB Pellizzari EH Canepa DF Cigorraga SB Possible involvement of ceramide in the regulation of rat Leydig cell function J Steroid Biochem Mol Biol 2000 75 4–5 307 313 11282287 10.1016/s0960-0760(00)00188-6

[B24] Hannun YA Functions of ceramide in coordinating cellular responses to stress Science 1996 274 5294 1855 1859 10.1126/science.274.5294.1855 8943189

[B25] Hammer S Sauer B Spika I Schraut C Kleuser B Schafer-Korting M Glucocorticoids mediate differential anti-apoptotic effects in human fibroblasts and keratinocytes via sphingosine-1-phosphate formation J Cell Biochem 2004 91 4 840 851 10.1002/jcb.10766 14991774

[B26] Nieuwenhuis B Luth A Chun J Huwiler A Pfeilschifter J Schafer-Korting M Kleuser B Involvement of the ABC-transporter ABCC1 and the sphingosine 1-phosphate receptor subtype S1P(3) in the cytoprotection of human fibroblasts by the glucocorticoid dexamethasone J Mol Med 2009 87 6 645 657 10.1007/s00109-009-0468-x 19370318

[B27] Sukocheva O Wadham C Holmes A Albanese N Verrier E Feng F Bernal A Derian CK Ullrich A Vadas MA Xia P Estrogen transactivates EGFR via the sphingosine 1-phosphate receptor Edg-3: the role of sphingosine kinase-1 J Cell Biol 2006 173 2 301 310 10.1083/jcb.200506033 16636149 PMC2063820

[B28] Aoki S Yatomi Y Ohta M Osada M Kazama F Satoh K Nakahara K Ozaki Y Sphingosine 1-phosphate-related metabolism in the blood vessel J Biochem 2005 138 1 47 55 10.1093/jb/mvi100 16046448

[B29] Argraves KM Argraves WS HDL serves as a S1P signaling platform mediating a multitude of cardiovascular effects J Lipid Res 2007 48 11 2325 2333 10.1194/jlr.R700011-JLR200 17698855

[B30] Kimura T Tomura H Mogi C Kuwabara A Damirin A Ishizuka T Sekiguchi A Ishiwara M Im DS Sato K Murakami M Okajima F Role of scavenger receptor class B type I and sphingosine 1-phosphate receptors in high density lipoprotein-induced inhibition of adhesion molecule expression in endothelial cells J Biol Chem 2006 281 49 37457 37467 10.1074/jbc.M605823200 17046831

[B31] Nofer JR van der Giet M Tölle M Wolinska I von Wnuck Lipinski K Baba HA Tietge UJ Gödecke A Ishii I Kleuser B Schäfers M Fobker M Zidek W Assmann G Chun J Levkau B DL induces NO-dependent vasorelaxation via the lysophospholipid receptor S1P3 J Clin Invest 2004 113 4 569 581 14966566 10.1172/JCI18004 PMC338256

[B32] Argraves KM Gazzolo PJ Groh EM Wilkerson BA Matsuura BS Twal WO Hammad SM Argraves WSHigh density lipoprotein-associated sphingosine 1-phosphate promotes endothelial barrier function J Biol Chem 2008 283 36 25074 25081 10.1074/jbc.M801214200 18606817 PMC2529014

[B33] Christoffersen C Ahnström J Axler O Christensen EI Dahlbäck B Nielsen LB The signal peptide anchors apolipoprotein M in plasma lipoproteins and prevents rapid clearance of apolipoprotein M from plasma J Biol Chem 2008 283 27 18765 18772 10.1074/jbc.M800695200 18460466

[B34] Christoffersen C Jauhiainen M Moser M Porse B Ehnholm C Boesl M Dahlbäck B Nielsen LB Effect of apolipoprotein M on high density lipoprotein metabolism and atherosclerosis in low density lipoprotein receptor knock-out mice J Biol Chem 2008 283 4 1839 1847 18006500 10.1074/jbc.M704576200

[B35] Christoffersen C Obinata H Kumaraswamy SB Galvani S Ahnström J Sevvana M Egerer-Sieber C Muller YA Hla T Nielsen LB Dahlbäck B Endothelium-protective sphingosine-1-phosphate provided by HDL-associated apolipoprotein M Proc Natl Acad Sci USA 2011 108 23 9613 9618 10.1073/pnas.1103187108 21606363 PMC3111292

[B36] Roy AK Lavrovsky Y Song CS Chen S Jung MH Velu NK Bi BY Chatterjee B Regulation of androgen action Vitam Horm 1999 55 309 352 9949684 10.1016/s0083-6729(08)60938-3

[B37] Heinlein CA Chang C The roles of androgen receptors and androgen-binding proteins in nongenomic androgen actions Mol Endocrino 2002 16 10 2181 2187 10.1210/me.2002-0070 12351684

[B38] Boonyaratanakornkit V Edwards DP Receptor mechanisms mediating non-genomic actions of sex steroids Semin Reprod Med 2007 25 3 139 153 10.1055/s-2007-973427 17447204

[B39] Migliaccio A Castoria G Di Domenico M de Falco A Bilancio A Lombardi M Barone MV Ametrano D Zannini MS Abbondanza C Auricchio F Steroid-induced androgen receptor-oestradiol receptor beta-Src complex triggers prostate cancer cell proliferation EMBO J 2000 19 20 5406 5417 10.1093/emboj/19.20.5406 11032808 PMC314017

[B40] Kousteni S Bellido T Plotkin LI O’Brien CA Bodenner DL Han L Han K DiGregorio GB Katzenellenbogen JA Katzenellenbogen BS Roberson PK Weinstein RS Jilka RL Manolagas SC Nongenotropic, sex-nonspecific signaling through the estrogen or androgen receptors: dissociation from transcriptional activity Cell 2001 104 5 719 730 11257226

[B41] Guo Z Benten WP Krucken J Wunderlich F Nongenomic testosterone calcium signaling Genotropic actions in androgen receptor-free macrophages J Biol Chem 2002 277 33 29600 29607 10.1074/jbc.M202997200 12048191

[B42] Sun M Yang L Feldman RI Sun XM Bhalla KN Jove R Nicosia SV Cheng JQ Activation of phosphatidylinositol 3-kinase/Akt pathway by androgen through interaction of p85alpha, androgen receptor, and Src J Biol Chem 2003 278 44 42992 43000 10.1074/jbc.M306295200 12933816

[B43] Nguyen TV Yao M Pike CJ Androgens activate mitogen-activated protein kinase signaling: role in neuroprotection J Neurochem 2005 94 6 1639 1651 10.1111/j.1471-4159.2005.03318.x 16011741

[B44] Sun YH Gao X Tang YJ Xu CL Wang LH Androgens induce increases in intracellular calcium via a G protein-coupled receptor in LNCaP prostate cancer cells J Androl 2006 27 5 671 678 10.2164/jandrol.106.000554 16728719

[B45] Zhang Y Chen CJ Yang QL Cheng LQ Wang H Huang LZ Effect of interfering hepatocyte nuclear factor-1 alfa in HepG2 on the expressions of apoM, apoA-I and the correlative key enzyme of cholesterol metabolism Zhonghua Gan Zang Bing Za Zhi 2011 19 2 121 126 21492516 10.3760/cma.j.issn.1007-3418.2011.02.012

[B46] Mosialou I Zannis VI Kardassis D Regulation of human apolipoprotein M gene expression by orphan and ligand-dependent nuclear receptors J Biol Chem 2010 285 40 30719 30730 10.1074/jbc.M110.131771 20660599 PMC2945566

[B47] Zhang X Zhu Z Luo G Zheng L Nilsson-Ehle P Xu N Liver X receptor agonist downregulates hepatic apoM expression in vivo and in vitro Biochem Biophys Res Commun 2008 371 1 114 117 10.1016/j.bbrc.2008.04.017 18413148

[B48] Bakker W Eringa EC Sipkema P van Hinsbergh VW Endothelial dysfunction and diabetes: roles of hyperglycemia, impaired insulin signaling and obesity Cell Tissue Res 2009 335 1 165 189 10.1007/s00441-008-0685-6 18941783

[B49] Yanagawa T Taniguchi A Fukushima M Nakai Y Nagasaka S Ohgushi M Matsumoto K Kuroe A Ohya M Seino Y Leptin, triglycerides, and interleukin 6 are independently associated with C-reactive protein in Japanese type 2 diabetic patients Diabetes Res Clin Pract 2007 75 1 2 6 10.1016/j.diabres.2006.04.019 16764962

[B50] Wannamethee SG Tchernova J Whincup P Lowe GD Kelly A Rumley A Wallace AM Sattar N Plasma leptin: associations with metabolic, inflammatory and haemostatic risk factors for cardiovascular disease Atherosclerosis 2007 191 2 418 426 10.1016/j.atherosclerosis.2006.04.012 16712853

[B51] Reilly MP Iqbal N Schutta M Wolfe ML Scally M Localio AR Rader DJ Kimmel SE Plasma leptin levels are associated with coronary atherosclerosis in type 2 diabetes J Clin Endocrinol Metab 2004 89 8 3872 3878 10.1210/jc.2003-031676 15292320

[B52] Luo G Hurtig M Zhang X Nilsson-Ehle P Xu N Leptin inhibits apolipoprotein M transcription and secretion in human hepatoma cell line, HepG2 cells Biochim Biophys Acta 2005 1734 2 198 202 10.1016/j.bbalip.2005.02.005 15904876

[B53] Xu N Ahrén B Jiang J Nilsson-Ehle P Down-regulation of apolipoprotein M expression is mediated by phosphatidylinositol 3-kinase in HepG2 cells Biochim Biophys Acta 2006 1761 2 256 260 10.1016/j.bbalip.2006.02.002 16542871

